# Quantitative Trait Locus and Brain Expression of HLA-DPA1 Offers Evidence of Shared Immune Alterations in Psychiatric Disorders

**DOI:** 10.3390/microarrays5010006

**Published:** 2016-03-07

**Authors:** Ling Z. Morgan, Brandi Rollins, Adolfo Sequeira, William Byerley, Lynn E. DeLisi, Alan F. Schatzberg, Jack D. Barchas, Richard M. Myers, Stanley J. Watson, Huda Akil, William E. Bunney, Marquis P. Vawter

**Affiliations:** 1 Functional Genomics Laboratory, Department of Psychiatry & Human Behavior, University of California, Irvine, CA 92697; lmorgan@uci.edu (L.Z.M.); bgalke@uci.edu (B.R.); psequeir@uci.edu (A.S.); 2 Department of Psychiatry & Human Behavior, University of California, Irvine, CA 92697, USA; webunney@uci.edu; 3 Department of Psychiatry, University of California, San Francisco, CA 94103, USA; William.Byerley@ucsf.edu; 4 VA Boston Healthcare System, Brockton, MA 02301, USA; DeLisi76@aol.com; 5 Department of Psychiatry, Harvard Medical School, Boston, MA 02115, USA; 6 Department of Psychiatry and Behavioral Sciences, Stanford University, Stanford, CA 94304, USA; afschatz@stanford.edu; 7 Department of Psychiatry, Cornell University of California, Ithaca, NJ 10065, USA; jbarchas@med.cornell.edu; 8 HudsonAlpha Institute for Biotechnology, Huntsville, AL 35806, USA; rmyers@hudsonalpha.org; 9 The Molecular & Behavioral Neuroscience Institute, University of Michigan, Ann Arbor, MI 48109, USA; watsons@umich.edu (S.J.W.); akil@umich.edu (H.A.)

**Keywords:** expression quantitative trait locus, major histocompatibility locus II, exon array, alternative splicing

## Abstract

Genome-wide association studies of schizophrenia encompassing the major histocompatibility locus (MHC) were highly significant following genome-wide correction. This broad region implicates many genes including the MHC complex class II. Within this interval we examined the expression of two MHC II genes (HLA-DPA1 and HLA-DRB1) in brain from individual subjects with schizophrenia (SZ), bipolar disorder (BD), major depressive disorder (MDD), and controls by differential gene expression methods. A third MHC II mRNA, CD74, was studied outside of the MHC II locus, as it interacts within the same immune complex. Exon microarrays were performed in anterior cingulate cortex (ACC) in BD compared to controls, and both HLA-DPA1 and CD74 were decreased in expression in BD. The expression of HLA-DPA1 and CD74 were both reduced in hippocampus, amygdala, and dorsolateral prefrontal cortex regions in SZ and BD compared to controls by specific qPCR assay. We found several novel HLA-DPA1 mRNA variants spanning HLA-DPA1 exons 2-3-4 as suggested by exon microarrays. The intronic rs9277341 SNP was a significant cis expression quantitative trait locus (eQTL) that was associated with the total expression of HLA-DPA1 in five brain regions. A biomarker study of MHC II mRNAs was conducted in SZ, BD, MDD, and control lymphoblastic cell lines (LCL) by qPCR assay of 87 subjects. There was significantly decreased expression of HLA-DPA1 and CD74 in BD, and trends for reductions in SZ in LCLs. The discovery of multiple splicing variants in brain for HLA-DPA1 is important as the HLA-DPA1 gene is highly conserved, there are no reported splicing variants, and the functions in brain are unknown. Future work on the function and localization of MHC Class II proteins in brain will help to understand the role of alterations in neuropsychiatric disorders. The HLA-DPA1 eQTL is located within a large linkage disequilibrium block that has an irrefutable association with schizophrenia. Future tests in a larger cohort are needed to determine the significance of this eQTL association with schizophrenia. Our findings support the long-held hypothesis that alterations in immune function are associated with the pathophysiology of psychiatric disorders.

## 1. Introduction

Alterations in immune function and reactivity in schizophrenia (SZ) and mood disorders have been found and extensively reviewed [1,2,3]. Reports of immune dysfunction in transcriptomic and proteomic studies of brain tissue have been reported primarily in SZ (reviewed in [4,5]). The majority of studies have analyzed antibodies, cytokines, and blood cell types, and indexed these markers as either an indicator of pro- or anti-inflammatory reactions or secondary responses. These studies have shown a remarkable diversity of findings and some gains in understanding relationships between psychopathology and immune function. For example, in schizophrenia, a meta-analysis of cytokine studies showed some consensus in acute relapse and first-episode psychosis for some cytokines (IL-1β, IL-6, and TGF-β), which might be state markers for acute exacerbations, whereas others (IL-12, IFN-γ, TNF-α, and sIL-2R) may be trait markers. One overview of these findings is that perhaps a subset of patients with psychopathology can be found with alterations in immune response [6]. Cell-type specificity was also analyzed and there were increases in markers for natural killer cells and CD4^+^ lymphocytes in schizophrenia [1]. More strikingly, some forms of autoimmune illness involve antibodies to glutamatergic receptors in brain (for review [7]) and are associated with both encephalitis and psychiatric disorders.

Epidemiological studies implicated immune mechanisms as risk factors for developing schizophrenia in offspring (recently reviewed in [8]). Some of the epidemiological studies have found a large odds ratio for factors such as season of birth, maternal viral infection, cytokine alterations, and antibody/viral titer for developing schizophrenia or other psychotic disorders. Other studies point to endogenous biomolecules, such as human endogenous retroviruses (HERV) products, to be possible underlying antigens that could be made in brain cells and trigger immune responses [9,10,11].

### 1.1. Genetic Loci Related to Major Histocompatibility (MHC) Region

Multiple genome-wide association (GWAS) studies have reported significant MHC single-nucleotide polymorphisms (SNPs) associated with schizophrenia, bipolar disorder, cognition, or hippocampal volume [12,13,14,15,16,17,18]. Finding highly associated SNPs within this region is a first step towards locating causative gene(s). Currently, these associations with schizophrenia do not necessarily imply immunity as the causative mechanism underlying the association, as the linkage disequilibrium is complicated across different populations. This highly replicated region encompasses hundreds of genes, some that are non-immune, in long-range linkage disequilibrium (LD), with extensive allelic heterogeneity. The GWAS studies to date have not found the gene(s) responsible or causative variants in the MHC locus, although the HLA-B and HLA-DRB1 loci were protective, and the HLA-C increased risk for schizophrenia [12]. One recent study of the C4 gene in the MHC locus showed an association with increased brain expression and risk of schizophrenia [19]. Thus, additional evidence of gene expression in the MHC locus needs to be pursued to directly examine some of the immune genes from persons with psychiatric disorders. 

### 1.2. Gene Expression in Schizophrenia, Major Depression, and Bipolar Disorder

The correlated expression of genes in brain or in peripheral tissues forms a co-expression network. Gene−gene expression correlation matrices can be built into coherent network connections. An expression quantitative trait locus (eQTL) study of a developing brain series mapped the significant loci to co-expression networks that were highly enriched for significant GWAS findings in schizophrenia [20]. The co-expression network was formed around a hub of several interconnected MHC genes, increasing interest to find the truly causative genes, and not merely candidate genes.

A second gene expression co-network of a module of genes that was differentially expressed between SZ and controls in whole blood also formed a network involving the MHC locus [20]. This co-expression network module was independent of antipsychotic medication in SZ subjects, and included multiple members of the MHC locus: heat shock protein 90 kDa α (cytosolic), class B member 1 (HSP90AB1); ring finger protein 1 (RING1); casein kinase 2, β polypeptide (CSNK2B); tubulin, β (TUBB); and ATP-binding cassette, sub-family F, member 1 (ABCF1). These two studies show the feasibility of expression studies to inform existing GWASes of differential regulation in brain and whole blood.

Microarray, quantitative PCR (qPCR) and RNA (cDNA) sequencing are useful techniques that examine gene expression in biological samples. The experiments that are reported begin with an inquiry into bipolar disorder using Affymetrix human exon 1.0 ST microarrays, to compare a matched cohort of bipolar type I subjects to controls using anterior cingulate cortex postmortem samples. The anterior cingulate is a region profoundly implicated in mood dysregulation. To validate the exon microarray results, qPCR experiments were performed in a larger cohort using five different brain regions, cell lines, and blood samples.

### 1.3. MHC Class II Genes

**Figure 1:**
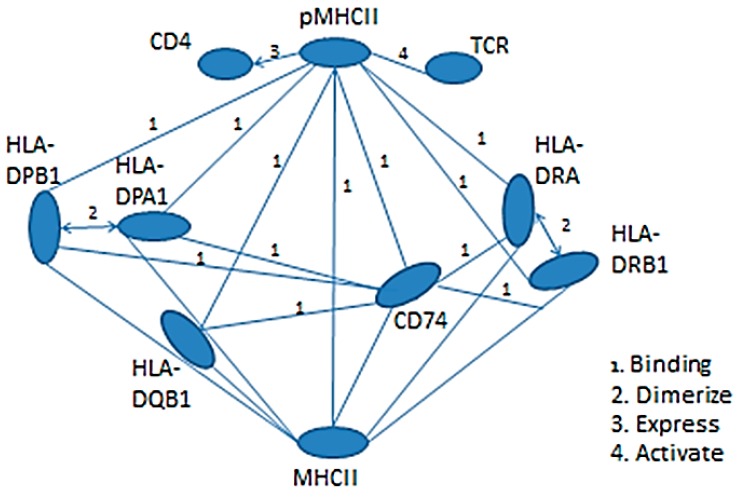
The interactions and molecular functions of three MHC Class II genes within the immune system involves antigen processing and presentation.

The purpose of the current study was to examine immune gene expression focusing on CD74, HLA-DRB1, and HLA-DPA1, which are integral components of the MHC Class II pathway (Figure 1). Using a cohort of postmortem brain tissue, we compared three diagnoses (schizophrenia, bipolar disorder type I, and major depressive disorder) to controls.

The three MHC Class II genes of relevance for this paper share well-described molecular functions within the immune system of antigen processing and presentation (Figure 1). CD74 has multiple roles in the immune system: it is a transmembrane protein that associates to MHC Class II α and β chains, and is a receptor of the proinflammatory cytokine macrophage migration inhibitory factor which modulates macrophage and monocyte activation. HLA-DPA1 dimers associate with CD74 trimers forming a heterononamer from NCBI RefSeq. The level of HLA-DRB1 gene expression is complex due to the multiple alleles and involvement of polymorphisms of the HLA-DRB1 promoter. Susceptibility to rheumatoid arthritis (RA) is associated with defined HLA-DRB1 alleles that are related to expression, and there is thought to be a negative correlation between incidences of RA and schizophrenia [21,22].

HLA-DPA1 SNPs, in particular rs3077, are associated with risk for persistent infection with the hepatitis B and C viruses, first noted in [23] and then subsequently replicated in multiple reports. Presumably, carriers of HLA-DPA1 coding variants along with variants in HLA-DPB and HLA-DRB have different sensitivities to viral antigen binding, clearing the virus by altering the antigen-presenting capabilities, and building effective immune response.

Taken together, the MHC Class II genes have multiple alleles and tissue-specific expression patterns which have not been described, and these transcripts have been quantitatively assessed in brain. Previously, MHC Class I genes were shown to be localized to neurons [24], while the expression of MHC Class II molecules in human brain has been found on microglia cells using OX-6 immunohistochemistry. Thus, we focused an investigation on members of the MHC Class II genes in multiple brain regions and disorders.

The MHC Class II are heterodimers composed of α and β transmembrane chains, such as HLA-DPA1 and HLA-DPB1, the latter a dimer that associates with the invariant chain of CD74 which forms a trimer in the endoplasmic reticulum. The trimer traffics to the Golgi compartment and then is transported to the late endosomic compartments, thereby loading the antigen peptide [25]. CD74 acts as a chaperone that regulates antigen presentation. Invariant chain of CD74 involves binding to the HLA-DPA1 and HLA-DPB1 dimers. After trafficking and targeting antigen, the invariant chain is released from MHC-II and then the antigen peptide binds [26,27]. The antigen-bound MHC-II complex moves to the plasma membrane. The plasma membrane-bound antigen-MHC II (pMHCII) interacts with T-cell receptor (TCR). TCR recognizes the antigen only when presented by foreign MHC molecules. TCR activates the pMHCII complex, and facilitate CD4 T-cell stimulation on the cell surface [28]. Crystal structure shows the ternary model of CD4-pMHCII-TCR that appears V-shaped with pMHC II at apex, but CD4 and TCR do not have direct contact. The signaling of CD4 and TCR is coordinated around the pMHCII [29]. Therefore, MHCII genes play an essential role in immune responses.

## 2. Methods

### 2.1. Exon Array (Experiment 1)

Anterior cingulate cortex (ACC) samples were used in an exon array (Affymetrix HuEx 1.0 ST, Santa Clara, CA, USA. ACC samples from 9 bipolar disorder patients and 11 healthy controls were used in this study for initial microarray analysis. The demographics are shown in Table 1. Approximately 100 mg of dissected frozen tissue was homogenized using Trizol Reagent (Invitrogen, Carlsbad, CA, USA) following the standard RNA Trizol isolation procedure: 1 mL Trizol was added to frozen brain, then Trizol mixture was homogenized for 30 s twice at 7500 rpm using Tissue Tearor (Biospec Products, Inc., Bartlesville, OK, USA) in ice. The mixture was subsequently incubated at room temperature for 5 min, 200 µL of chloroform added, the tube shaken by hand for 30 s and the mixture then incubated for 2–3 min at reverse transcription (RT), before being centrifuged at 12,000 *g* for 15 min at 4 °C with the Eppendorf Centrifuge 5417R (Eppendorf, Hauppauge, NY, USA). The supernatant containing the upper aqueous phase was transferred to a new tube, mixed with 500 µL of isopropyl alcohol and incubated for 15 min at RT and centrifuged at 12,000 *g* for 10 min at 4 °C. The supernatant was removed, and the pellet was washed with 1 mL of iced 75% ethanol, by brief vortex, then centrifuged at 7500 *g* for 10 min at 4 °C. Ethanol was decanted, and RNA pellet was dried at RT for 5–10 min in a laboratory hood by opening tube lid; RNA was then dissolved in 50 µL DEPC-treated water by gently mixing on ice. RNA was stored in a −80 °C freezer. The resulting total RNA was cleaned of low molecular weight fragments by passing through a Qiagen column, and checked on an Agilent 2100 Bioanalyzer (Agilent Technologies, Santa Clara, CA, USA) for quality control using RNA integrity number. The concentrations measured on a spectrophotometer (Molecular Devices, Sunnyvale, CA, USA) were adjusted to 1 µg/µL.

GeneChip Whole Transcript Sense Assay Protocol:

The Affymetrix Human GeneChip Exon 1.0 ST arrays were run following the manufacturer’s protocol (Affymetrix, Santa Clara, CA, USA). Briefly, 2 μg of purified total RNA underwent ribosomal RNA removal using the RiboMinus Human/Mouse Transcriptome Isolation Kit (Invitrogen). The reduced RNA was then reverse transcribed to cDNA using random hexamers tagged with a T7 promoter sequence followed by a second strand cDNA synthesis using DNA polymerase (GeneChip WT cDNA Synthesis and Amplification Kit, Affymetrix). The resulting double-stranded cDNA was then used for amplification of antisense cRNA using the Gene Chip Sample Cleanup Module (Affymetrix). A second cycle cDNA synthesis was performed using random primers to reverse transcribe the cRNA into sense single-stranded DNA. The DNA was then enzymatically fragmented and labeled using the GeneChip WT Terminal Labeling Kit (Affymetrix). The hybridization cocktail consisting of the labeled sample, Control Oligonucleotide B2, and 20× Eukaryotic Hybridization Controls were heated for 5 min at 99 °C and cooled for 5 min at 45 °C, then centrifuged 1 min. A volume of 200 µL was loaded onto the Affymetrix Human Gene Chip Exon 1.0 ST Arrays and the arrays were placed in a 45 °C hybridization oven, at 60 rpm, to incubate for 17 h. The GeneChip Hybridization, Wash and Stain Kit (Affymetrix) was used with the Fluidics Station 450_0001 protocol. Arrays were then scanned on the GeneChip Scanner 3000 7G (Affymetrix). All exon array samples were processed in the same batch by one person.

Each CEL file from the Affymetrix HuEx 1.0 ST was imported into Partek Genomics Suite 6.6 using background subtraction and elimination of probes with common SNPs from analysis of exon array data following the method of Gamazon *et al.* 2010 [30]. The initial count of probesets on the array is 1.1 million (see Supplementary Methods), elimination of common SNPs in probes reduced the probeset count by ~350,000 probesets. Each CEL file was analyzed together using robust multiarray analysis (RMA) [31]. Resulting expression values of probes were averaged in each probeset. Each probeset was aligned to a unique RefSeq gene, and we report only findings that are associated with full-length mRNA and have coverage by at least two probesets. This reduced the total probesets analysis to 230,659 probesets representing 11,807 full-length RefSeq genes. The diagnosis by probeset interaction was calculated in Partek (Supplementary Methods), and the interaction *p*-values cut-off were determined after Bonferroni correction.

### 2.2. qPCR (Experiment 2)

These same subjects in Experiment 1 were used for qPCR, plus ~90 additional subjects for Experiment 2 (shown in Supplementary Table 1). Total RNA was extracted from five brain regions (dorsolateral prefrontal cortex (DLPFC), amygdala, hippocampus, nucleus accumbens, and cerebellum) for each subject using the method outlined in Section 2.1. Total RNA from the DLPFC, amygdala, hippocampus, nucleus accumbens, and cerebellum were used for making complementary DNA (cDNA) (Table 2). cDNA was generated using TaqMan reverse-transcription (RT) reagents according to the manufacturer’s protocol (Applied Biosystems, Foster City, CA, USA), and cDNAs were aliquoted and stored at −20 °C. In brief, the cDNA synthesis contained 5 µL of 10× Taqman RT buffer; 11 µL of 25 mM MgCl_2_; 10 µL of deoxy NTPs; 2.5 µL of Oligo d(T)_16_ primer; 1 µL of RNase inhibitor; 1.25 µL of Multiscribe reverse transcriptase, and 1 µL of RNA (1 µg/µL). Two separate 50 µL reactions for each RNA were performed and combined together. Each cDNA batch reaction had a maximum of 24 tubes to ensure the best sample quality. The primers were designed using Primer Express software (Applied Biosystems) and purchased from Bioneer, Inc. Factors including melting temperature and guanine-cytosine (GC) content were considered. The HLA-DPA1 forward and reverse primers were designed to hybridize to sequences located in exon 3, near the site of hybridization for the probe (Probe Set 2950343; Affymetrix, Inc.). The primer set was BLAST searched against the entire human genomic sequence database for specificity, and primers used are shown in Supplementary Methods, Part 3 for all genes. The HLA-DPA1 primers were tested by using a set of cDNAs from cerebellum), genomic DNA, no template control (NTC), and RT minus (two individual DLPFC RNAs without cDNA). The primer test results showed that all cDNA amplified with a single band, while the NTC and gDNA amplified greater than 40. The RT minus showed greater than six cycles difference from the cDNA samples. This detection ensured that the HLA-DPA1 primers were specific to HLA-DPA1. Similar procedures were used for HLA-DRB1 and CD74 qPCR analyses.

Quantitative PCR (qPCR) was performed on an ABI 7900HT Sequence Detection System (Applied Biosystems) in 384-well plates. The samples were aliquoted by the Biomek3000 Robot (Beckman Coulter, Brea, CA, USA) and run in triplicate using one plate per gene. The reaction was performed in a 12.5 µL total volume with 6.25 µL of 2× SYBR Green Master Mix (Applied Biosystems); 0.25 µL of 10 µM forward primer; 0.25 µL of 10 µM reverse primer; 2 µL of a 1:10 dilution of cDNA template (corresponding to approximately 4 ng RNA), and water to a total volume of 12.5 µL. The thermal cycle conditions were: 50 °C for 2 min (incubation), 95 °C for 10 min (activation), 45 cycles at 95 °C for 15 s (denaturation), and 60 °C for 1 min (annealing/extension), and a final dissociation step at 95 °C for 15 s, 60 °C for 15 s, and 95 °C for 15 s. The qPCR cycle threshold (*C*t) was set in the middle of the exponential phase of the amplification. In each experiment, the individual sample was run in triplicate and the *C*t of each well was recorded at the end of the reaction. The mean and standard deviation (SD) of the three *C*ts were calculated and the average value was accepted if the triplicate *C*t values were within ±1 *C*t. Two representative qPCR runs of HLA-DPA1 robotically pipetted in 384-well assay plates were examined for two brain regions, and three wells were eliminated. The average coefficient of variation was ~0.8% for each plate. The relative quantification was used to measure gene expression. Glyceraldehyde-3-phosphate dehydrogenase (GAPDH) and succinate dehydrogenase complex subunit A (SDHA) were selected as the housekeeping genes. After correction with the mean of the two housekeeping genes (*C*t _target_ − *C*t _mean of housekeeping_), an ANCOVA was used for the average delta *C*t values for each subject. A repeated-subjects ANCOVA was used with factorial blocks of diagnosis, region, and SNP rs9277341, and also included age, RIN, and pH covariates. The fold change in gene expression was calculated to elucidate the direction of differences in mRNA levels between diagnosis and control samples in each brain region.

### 2.3. Alternative Splicing of HLA-DPA1 (Experiment 3)

By exon array, a variable expression pattern was observed across exons 2, 3, and 4 in HLA-DPA1 for multiple subjects. This gene was therefore chosen for analysis of alternative splicing by direct sequencing of gel-purified cDNA amplicons to evaluate potential variants.

Representative cDNA samples from subjects in Experiment 2 were PCR amplified and run on agarose gels for separation of bands. cDNA amplification was conducted from exons 2 through 4, to screen for alternative splicing. Gel bands smaller than full length were visualized on gels, and sent for Sanger sequencing. The subjects screened are shown in Supplementary Table 1.

### 2.4. Lymphoblast Cell Line (LCL) qPCR Biomarker (Experiment 4)

In addition to testing brain samples by qPCR (Experiment 2), 87 EBV-transformed lymphoblast cell lines (LCLs) from Costa Rica were tested for expression levels of the three MHC Class II genes via qPCR. Previously transformed cell lines were grown to confluency and harvested for RNA using Trizol. cDNA was synthesized as described above (Table 3, demographics).

## 3. Results

### 3.1. MHC Class II Expression in Cingulate Cortex by Exon Array in Bipolar Disorder Compared to Controls (Experiment 1)

The data analysis for the Affymetrix Human Exon 1.0 ST array used an ANOVA with diagnosis and probesets as between subject factors. There were 11,807 transcript identifiers analyzed composed of 230,659 probesets. Affymetrix array transcript identifiers that passed this filter for diagnosis by probeset interaction are listed (Supplementary Tables 2–4), and there were 30 genes (29 known genes as ENSG00000185790 did not map to a known RefSeq gene) that passed Bonferroni multiple comparisons correction for diagnosis by probeset. The analysis was restricted to male control and bipolar patients to minimize heterogeneity of expression due to sex differences, as well as there being unequal and fewer females in the study. There were no genes that passed Bonferroni corrections for diagnosis factor.

**Figure 2:**
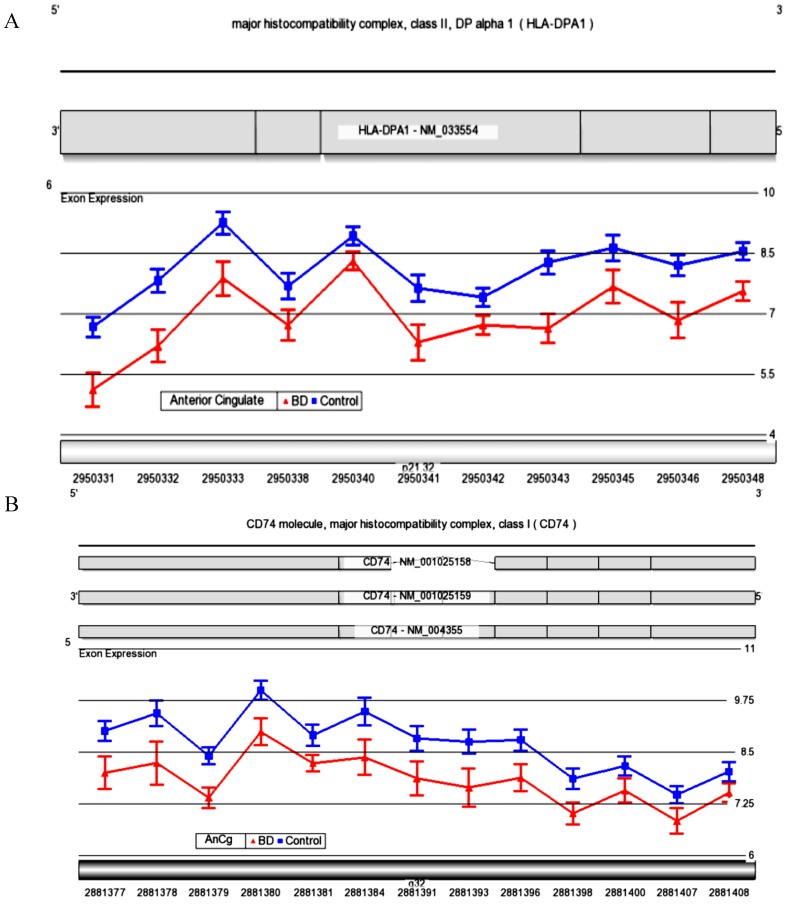
(**A**) HLA-DPA1 probeset shown on *x*-axis and probe intensity shown on *y*-axis in log_2_ scale. All probesets were significantly decreased (*p* < 0.05) in bipolar disorder (BD, red) compared to controls (C, blue), except two probesets (2950338 and 2950345) that were at trend level (*p* < 0.1). The *p*-values are shown in Table 4 for each probeset comparison; (**B**) The plot of CD74 for BD and C supports decreased expression along entire transcript.

Two MHC Class II genes (HLA-DPA1 and CD74) passed Bonferroni correction (Supplementary Tables 2–4). *Post hoc* analysis of HLA-DPA1 showed downregulation of HLA-DPA1 mRNA in the ACC of bipolar disorder patients relative to healthy control subjects using the Affymetrix Human Exon 1.0 ST array data (GEO Accession Number: GSE78246) (Table 4) along the entire length of the gene (Figure 2A). There was a statistically significant decrease in BD in ACC on HLA-DPA1 gene expression (*p* = 0.013); however, the interaction effect between diagnosis and probesets was highly significant (*p* = 1.6 × 10^−6^). The individual probesets in the exon array that were significantly decreased in bipolar subjects in ACC compared to controls were the following: Affymetrix probesets: 2950331, 2950332, 2950333, 2950336, 2950341, 2950342, 2950343, 2950346, 2950348 (Figure 2A). CD74 was significantly decreased in expression in BD compared to controls by exon array (*p* = 0.038, Figure 2B), and showed a highly significant interaction effect between diagnosis and probesets (*p* = 3.3 × 10^−5^). For the other MHC Class II transcript, HLA-DRB1, the transcript level was not altered in BD compared to controls (*p* = 0.27), nor was there a significant interaction effect.

**Figure 3:**
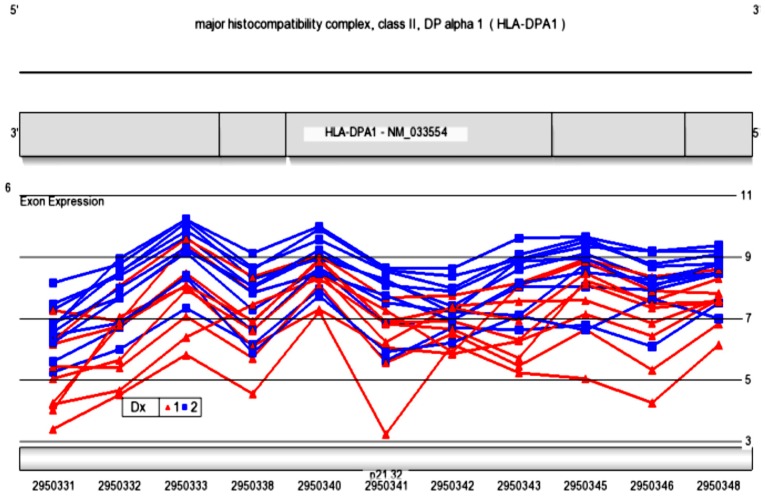
Individual samples for each probeset on HLA-DPA1 suggests differences within subjects consistent with a significant diagnosis by probeset interaction (*p* = 1.6 × 10^−6^).

**Figure 4:**
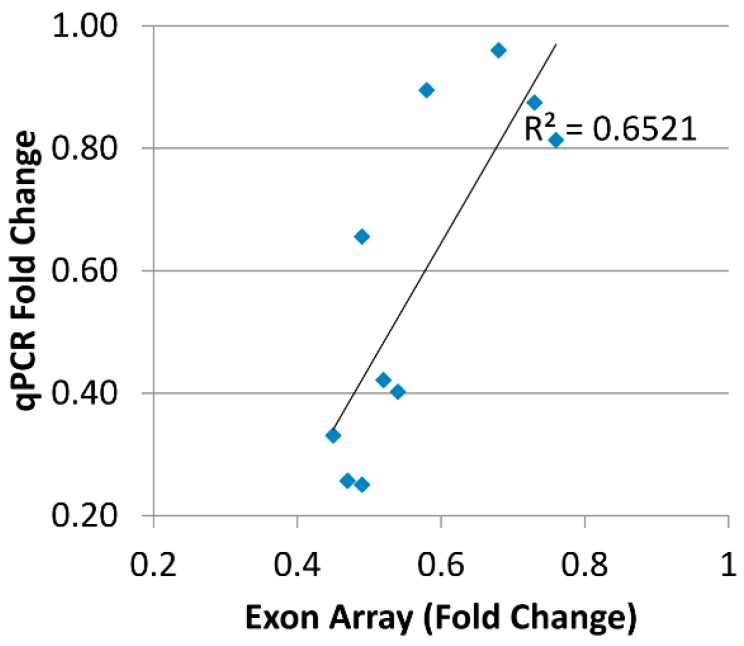
Comparison of fold change results for qPCR (*y*-axis) and Affymetrix exon array (*x*-axis) based upon genes shown in Table 5.

The HLA-DRB1 diagnosis by probeset was not significant (*p* = 0.93). A variable expression of HLA-DPA1 between subjects by exon array probesets (Figure 3) suggested that exons 2, 3, and 4 might be sites of alternative splicing, which were investigated by PCR sequencing (see below). For validation of the exon array results, we chose additional qPCR candidates based upon microarray results shown in Supplementary Table 1 of significant evidence of diagnosis by probeset. Our validations Figure 4 were consistent for microarray and exon array fold changes (*r* = 0.80).

The Affymetrix exon array fold changes were consistent with the result shown for exon array of anterior cingulate cortex.

### 3.2. qPCR Validation of Candidate MHC II Genes Identified in Exon Array Data (Experiment 2)

The main effects for all qPCR analyses are shown in Table 6. Age and pH were selected as covariates, while diagnosis, brain region, and sex were main effects. The ANCOVA for age was significant (HLA-DRB1 and HLA-DPA1 *p* < 0.0005, while CD74 was nominally significant *p* = 0.05). Including pH in the ANCOVA was not significantly related to gene expression, and sex was significant for HLA-DRB1 expression (*p* = 0.001).

**Figure 5:**
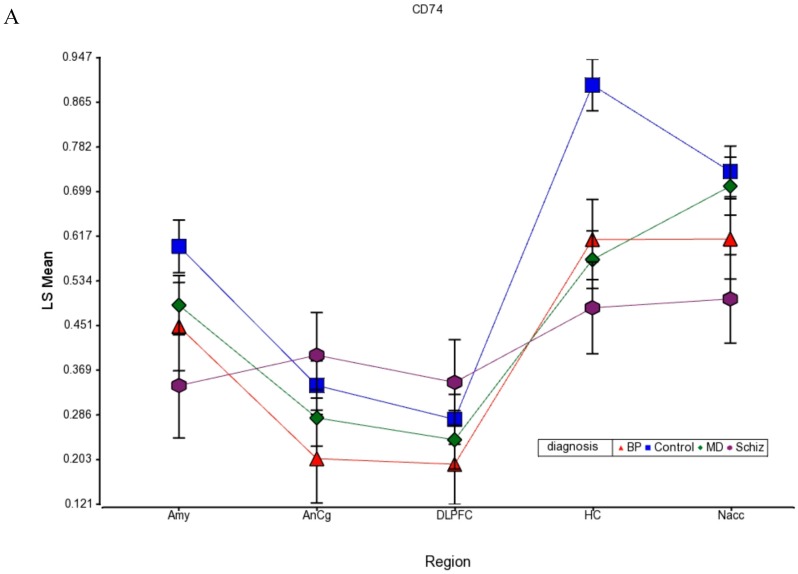

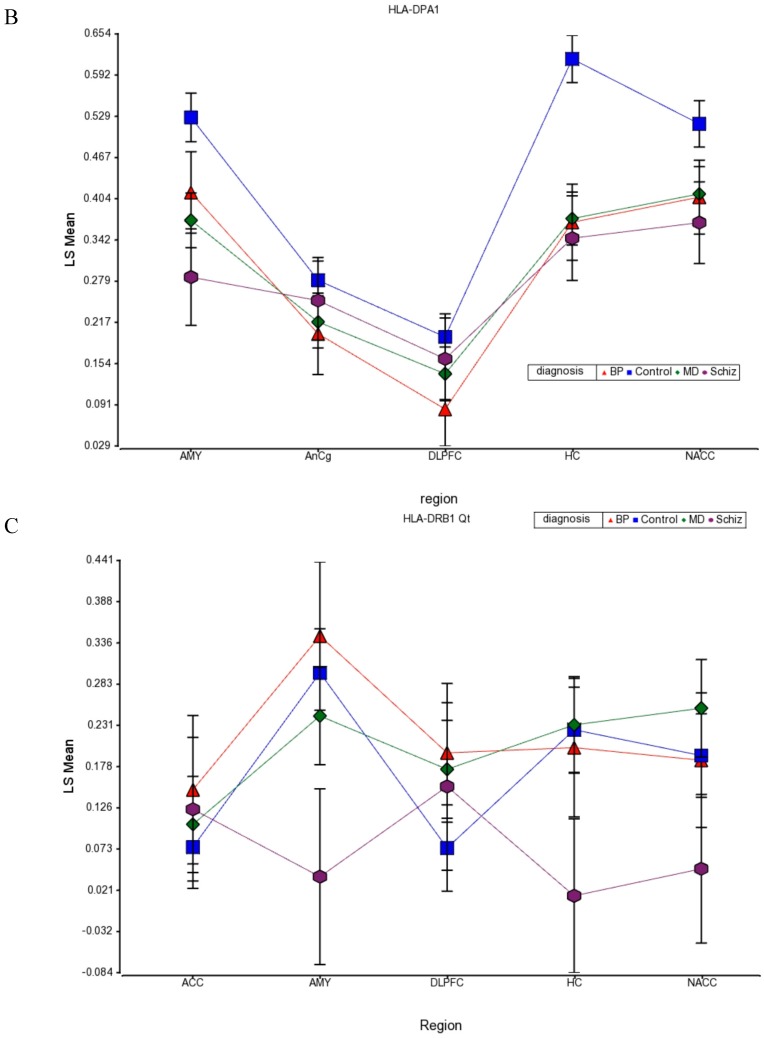
qPCR lineplot results of five brain regions (amygdala (AMY), anterior cingulate (ACC), dorsolateral prefrontal cortex (DLPFC), hippocampus (HC), and nucleus accumbens (NACC)) for three diagnoses (bipolar disorder, major depressive disorder, schizophrenia) compared to controls. The overall results show a decline in expression for CD74 (**A**) and HLA-DPA1 (**B**) across regions for neuropsychiatric disorders compared to controls. The statistics for the comparisons are shown in Table 7. HLA-DRB1 was not significant in comparisons with any of the groups (**C**).

Highly significant main effects were found for HLA-DPA1 and CD74 (*p*-values < 10^−20^), and the resulting line plots are shown in Figure 5. For diagnosis effect, CD74 (*p* = 3.6 × 10^−5^) and HLA-DPA1 (*p* = 1.7 × 10^−7^) were significant, while HLA-DRB1 did not reach significance (*p* = 0.078). Only CD74 was significant for region by diagnosis interaction. Regional analyses were conducted and comparison results are shown in Table 7 A,B,C. The lack of findings for HLA-DRB1 in all brain regions by qPCR is consistent with the exon microarray findings. Three neuropsychiatric disorder groups (SZ, MDD, and BD) had a significantly lower expression of HLA-DPA1 in the hippocampus. Further, CD74 and HLA-DPA1 showed concordant expression at the brain region level, suggesting broad co-expression. SZ showed reduced expression in similar regions as CD74 and HLA-DPA1. HLA-DRB1 showed a variable relationship across diagnosis and brain regions, and, due to higher cycle numbers, might not be reliably detected in brain, at least the isoform that we measured by qPCR.

### 3.3. Expression by Genotype Interaction of HLA-DPA1

The same cohort of subjects which had brain gene expression measured for HLA-DPA1 (Table 2) was genotyped for SNPs around the HLA-DPA1 gene and an F-ratio was calculated for association. The HLA-DPA1 expression levels were measured by qPCR for the association analysis with the genotypes of SNP rs9277341.This intronic SNP was located in intron two of HLA-DPA1. We sequenced the specific amplicon resulting from qPCR amplification reactions (Supplementary Table S1) to find the expected product and ensuring that SNPs or large exonic deletions were not within the primer region of the qPCR amplicon.

**Figure 6:**
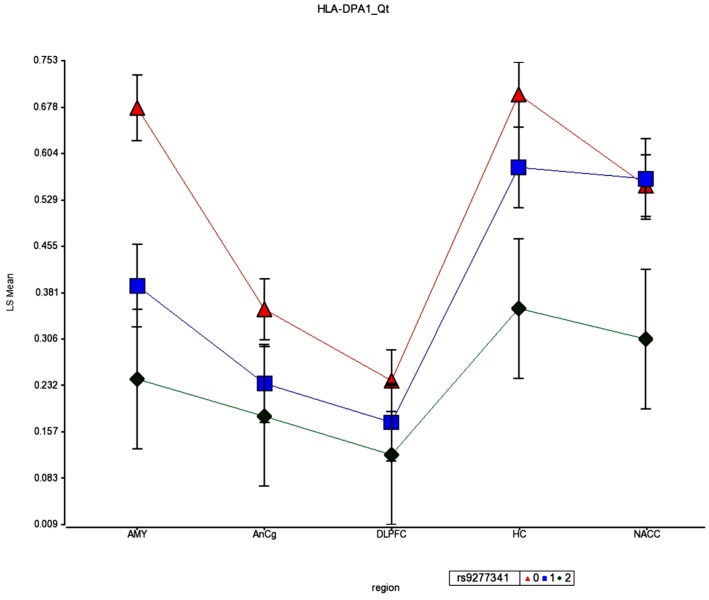
Genotype association analysis of HLA-DPA1 expression with rs9277341. The 0, 1, 2 groups represent the major allele, heterozygotes, and minor allele of SNP rs9277341. For the rs9277341, the SNP was sequenced and confirmed as (AA = 0), (AG = 1), and (GG = 2) on the negative strand. This corresponds with major and minor alleles, T (major) and C (minor) on the positive strand. The rs9277341 genotype main effect was significant for HLA-DPA1 expression (*p* = 0.0002) across regions, the effect of region x SNP rs9277341 was not significant, and region effect was highly significant (3.1 × 10^−17^).

The results for HLA-DPA1 SNP rs9277341 by brain region are shown (Figure 6). The main effect for rs9277341was significant (*p* = 0.0002), the major ‘T’ allele showed increased expression, while the homozygous minor “C” allele has significantly lower expression. The interaction of region (five brain regions tested) by SNP rs9277341 genotype (0, 1, 2) was not significant. The overall effect of diagnosis factor (SZ, BD, MDD, C) on HLA-DPA1 expression was significant (*p* = 1.68 × 10^−6^). *Post hoc* comparisons between each psychiatric diagnosis and controls were significant, and decreased significantly in expression (Table 8 and Table 9).

### 3.4. Alternative Splicing Validation HLA-DPA1 (Experiment 3)

**Figure 7:**
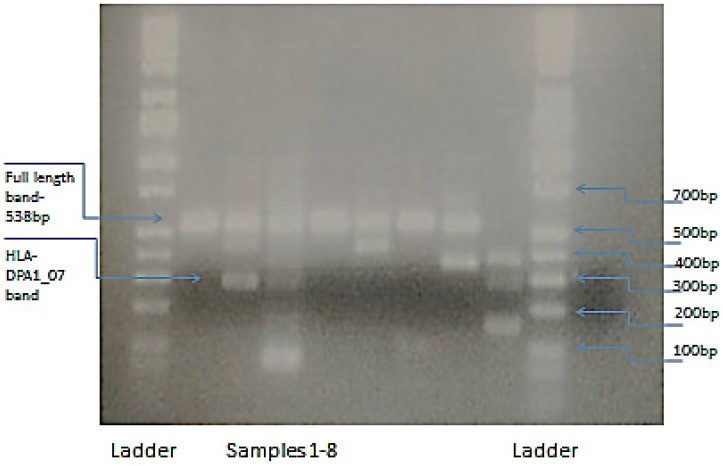
The gel images showed the multiple bands of HLA-DPA1 exon 2–4 from anterior cingulate brain samples. Full-length bands 538 bp and multiple bands of HLA-DPA1 exon 2–4 alternative splicing.

Gel electrophoresis of amplicons from cDNA for HLA-DPA1 exon 2–4 amplification showed the predominant expected band size of 538 bp. However, in many brain samples, distinct smaller bands were seen (Figure 7). These gel-purified bands were directly sequenced and the results confirmed alternative splicing of these smaller bands as containing parts of exons 2, 3, and 4 (Table 10). The HLA-DPA1 sequencing primers are located in exon 2 and exon 4 of NM_033554 as:

Forward5’-TGGACAAGAAGGAGACCGTCT-3’(positon307/327);

Reverse5’-TTTATGATGAGGACGGTGCC-3’(position 844/825). All the sequences were in the range from 307 to 844 of NM_033554.

**Figure 8:**
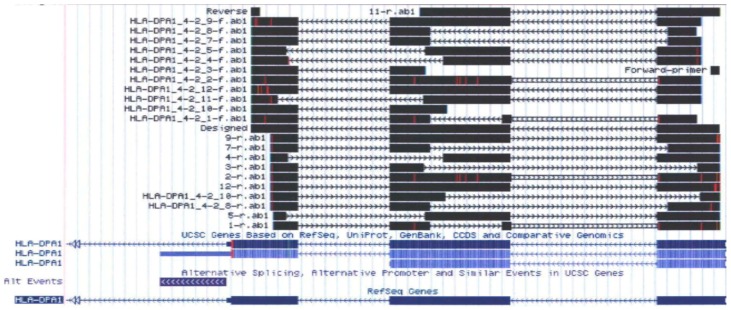
Alternative splicing of HLA-DPA1 in anterior cingulate cortex samples showing alignment of representative amplicons to RefSeq mRNA in UCSC Genome Browser. Forward and reverse sequences are aligned to RefSeq Genes using UCSC BLAT. The forward and reverse sequences are denoted with ‘f’ and ‘r’ in label, e.g., HLA-DPA1_4_2_9_f.ab1 is the same subject as 9-r.ab1.

The cDNA alignment to HLA-DPA1 RefSeq showed that differential splicing of exon 2–3 and 3–4 was involved, although did not involve exon skipping, tending rather toward a novel splice site extended into adjacent exons (Figure 8). The sequence of four subjects shows full-length amplicon of exons 2, 3, and 4 (512–515 bp), and truncated versions of exons 2, 3, and 4 (215–400 bp). The UCSC sequence tracks show truncation of exon 2, 3, and 4, thereby suggesting different cryptic splice sites that occur (Figure 8).

Total sequences from forward primer to reverse primer are 538 bp, and in position 307−844 of NM_033554.3. Exon Bound1aries 2 (264–453) 3 (454–735) 4 (736–902) representative samples from four different subjects using anterior cingulate cDNA. The forward primer sequences (-f.ab1 are matched to reverse primer sequence nomenclature (-r.ab1).

For more details, the next section shows the sequencing results for forward and reverse primers for HLA-DPA1_07 splicing site position from 427 to 641 in NM_033554 and the HLA-DPA1_01 splicing site at position 478 to 643 in NM_033554.

Box 1 **Sequencing Result of Bands from HLA-DPA1-07 Gel.**

**Sequencing of cDNA HLA-DPA1_ (07-f.ab1 with Forward primer)**
 
350AGCCTTTTCCTTTGAGGCTCAGGGCGGGCTGGCTAACATTGCTATATTGAACAACAACTTGAATACCTTGATCCAGCG427        (spliced        out         region)
---------------------------------------------------------------------------------------------------------------------------------------------642TTCCATTACCTGACCTTTGTGCCCTCAGCAGAGGACTTCTATGACTGCAGGGTGGAGCACTGGGGCTTGGACCAGCCGCTCCTCAAGCACTGGG735
736AGGCCCAAGAGCCAATCCAGATGCCTGAGACAACGGAGACTGTGCTCTGTGCCCTGGGCCTGGTGCTGGGCCTAGTCGGCATCATCGTGGGCACCGTCCTCATCATAAA844
 
**Sequencing of cDNA HLA-DPA1_(07-r.ab1 with Reverse primer)**
 
801GCACCnGGCCCAGGGCACAGAGCACAGTCTCCGTTGTCTCAGGCATCTGGATTGGCTCTTGGGCCT736
735CCCAGTGCTTGAGGAGCGGCTGGTCCAAGCCCCAGTGCTCCACCCTGCAGTCATAGA AGTCCTCTGCTGAGGGCACAAAGGTCAGGTAATGGAA642  (spliced out region)
---------------------------------------------------------------------------------------------------------------------------------------------427CGCTGGATCAAGGTATTCAAGTTGTTGTTCAATATAGCAATGTTAGCCAGCCCGCCCTGAGCCTCAAAGGAAAAGGCTTGGCCAAACTCCTCCAGATGCCAGACGGTCTCCTTCTTGTCCA307

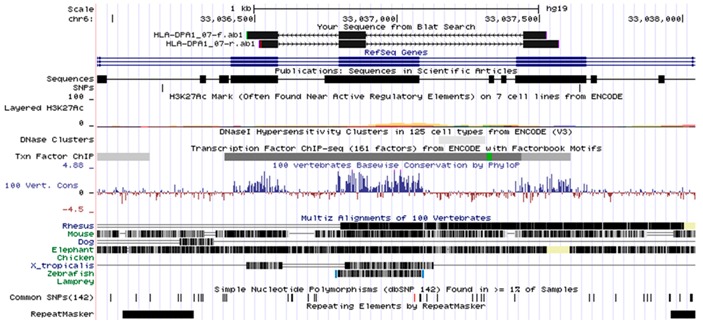

The sequencing result for both forward and reverse primers are consistent to show a novel splice site for HLA-DPA1 located in exons 3 and 4.

Box 2 **Sequencing Result of Bands from HLA-DPA1-01 Gel.**


cDNA HLA-DPA1_(01-f.ab1 with Forward primer).
360TTTGAGGCTCAGGGCGGGCTGGCTAACATTGCTATATTGAACAACAACTTGAATACCTT
GATCCAGCGTTCCAACCACACTCAGGCCGCCAATGATCCCCCTGAGGTGACCGTGTTTC478
(spliced                                                     out      
region)---------------------------------------------------------------------------------------------------------------------------------------------644CATTACCTGACCTTTGTGCCCTCAGCAGAGGACGTCTATGACTGCAGGGTGGAGCACTGGGGCTTGGACCAGCCGCTCCTCAAGCACTGGGAGGCCCAAGAGCCAATCCAGATGCCTGAGACAACGGAGACTGTGCTCTGTGCCCTGGGCCTGGTGCTGGGCCTAGTGGGCATCATCGTGGGCACCGTCCTCATCATAAA844
 
**cDNA HLA-DPA1_(01-r.ab1 Reverse primer)**
 
796GGCCCAGGGCACAGAGCACAGTCTCCGTTGTCTCAGGCATCTGGATTGGCTCTTGGGCCTCCCAGTGCTTGAGGAGCGGCTGGTCCAAGCCCCAGTGCTCCACCCTGCAGTCATAGACGTCCTCTGCTGAGGGCACAAAGGTCAGGTAATG644          (spliced                  out 
region)------------------------------------------------------------------------------------------------------------------------------------478 GAAACACGGTCACCTCAGGGGGATCATTGGCGGCCTGAGTGTGGTTGGAACGCTGGATCAAGGTATTCAAGTTGTTGTTCAATATAGCAATGTTAGCCAGCCCGCCCTGAGCCTCAAAGGAAAAGGCTCGGCCAAACTCCTCCAGATGCCAGACGGTCTCCTTCCTTGTCC307

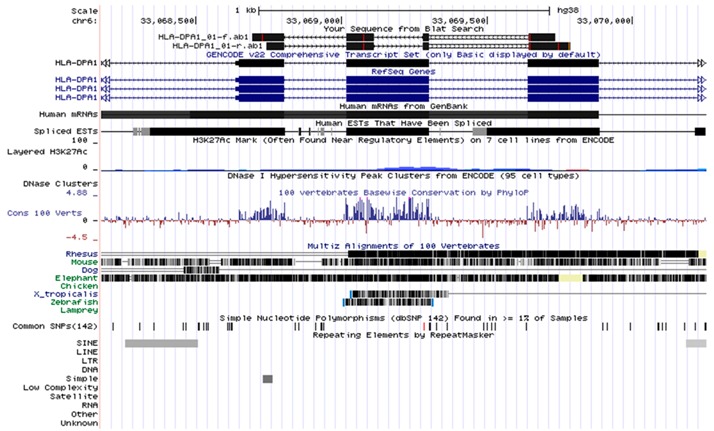

The sequencing result for both the forward and reverse primer shows a consistent alternative splice site in exon 3.

### 3.5. Potential Biomarker Analysis (Experiment 4)

Since these MHC Class II molecules are predominantly immune in function, we explored potential biomarkers using 87 EBV-transformed LCLs (Table 3, demographics) collected from Costa Rica. The four groups were closely matched in demographics. This sample was comprised of gender-and age-matched living subjects collected for a separate genetic study of cases and controls in Costa Rica [32]. The subjects are of Spanish ancestry, and admixed [33]. The samples of LCLs were grown in similar passages and timeframes in the lab (LM), using similar protocols for each cell line and RNA extraction. An ideal biomarker would focus on whole blood from subjects, instead of derived LCLs.

We tested expression of three MHC Class II genes for differential expression by qPCR. The results showed that CD74 and HLA-DPA1 were both significantly decreased in BD, and trends for a decrease in SZ were also found (Table 11). These findings were supportive of the decreases seen in different brain regions for these MHC Class II genes for BD and SZ. Interestingly, HLA-DRB1 was not different between the three groups, consistent with a lack of differences in MDD, SZ, or BD as shown by brain expression studies.

### 3.6. Blood−Brain Comparison

**Figure 9:**
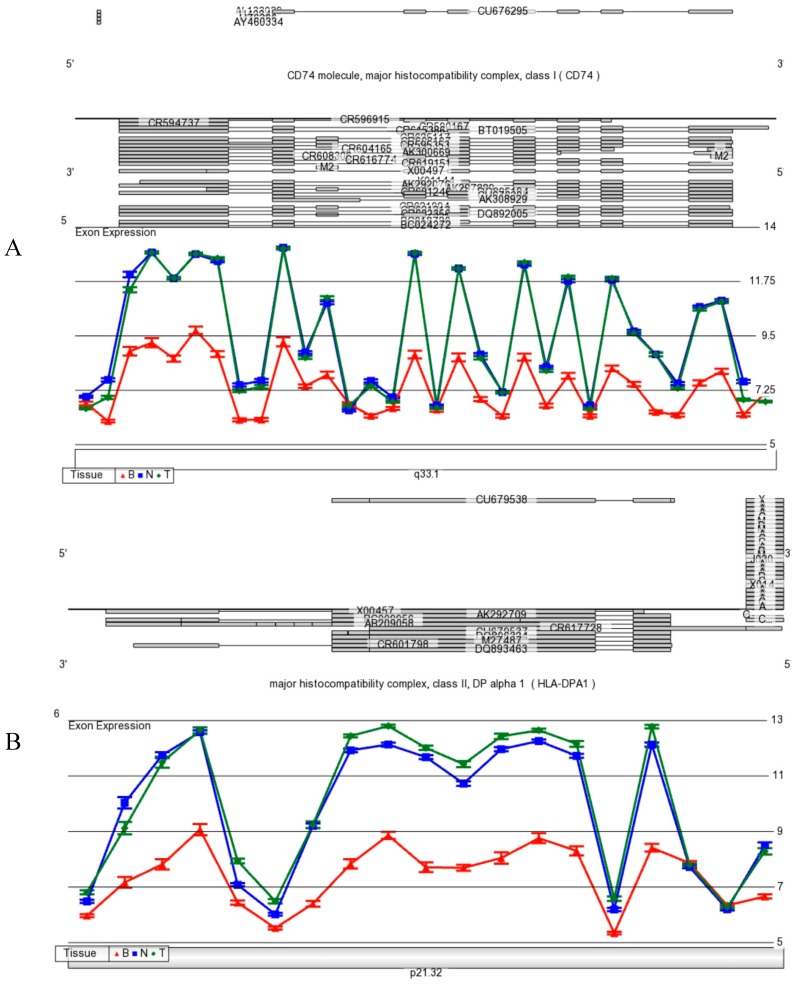

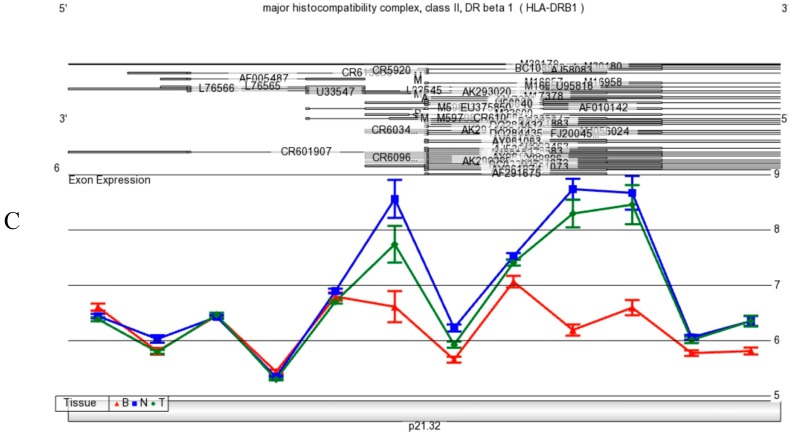
Exon microarray comparison of expression across brain samples (B), non-transformed whole blood samples and PBMC (N), and LCL EBV-transformed cell lines (T) for CD74 (Figure **A**); HLA-DPA1 (Figure **B**); and HLA-DRB1 (Figure **C**). In all three cases, the N and T levels (blood and immune cell levels) were higher than brain expression levels, and the higher expression levels by exon array were also confirmed by qPCR. The *x*-axis for each figure shows the probeset locations, and *y*-axis for each figure shows the probeset intensity in log_2_ scale.

We extracted from exon array analysis the three MHC II mRNA expression levels for HLA-DPA1, CD74, and HLA-DRB1 from 187 different samples that had been run in-house for a variety of experiments. Those samples are broadly categorized as brain (*n* = 29); whole blood and PBMC (*n* = 84); and LCLs (*n* = 74) (Table 12 shows details for each category). These three tissues were summarized for exon array expression (brain, transformed cell lines, and non-transformed cells) and the results are shown in Figure 9 A,B,C. As expected, both transformed and non-transformed cells show almost identical expression levels, and were significantly increased from 4–16 times higher expression compared to brain. To determine that residual blood levels were not affecting the brain levels measured by exon array, a series of three brains were measured by analogous rat exon array, with one brain cleared with saline rinse perfusion via carotid artery. The brain levels of three MHC Class II molecules were not altered by perfusion, thus indicating that brain levels, although lower than blood levels, were not the result of residual amounts of blood within the brain.

**Figure 10:**
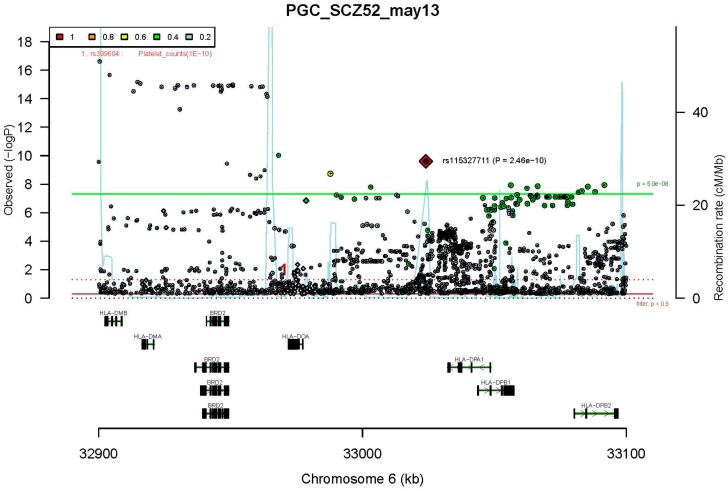
MHC region association to schizophrenia [18] shown for 32.9–33.1 Mb. A strongly associated SNP with schizophrenia (rs115327711) is located at chr6:33,024,031 (association *p* = 2.46 × 10^−10^, OR = 1.1956) and shown in large red triangle. The HLA-DPA1 eQTL rs9277341 is 15 kb upstream at chr6:33,039,625.

The HLA-DPA1 gene is located at the edge of the strong MHC association signal to schizophrenia [18] shown in Figure 10. A highly significant SNP associated with schizophrenia is 15 kB downstream of the eQTL for HLA-DPA1 and D’ is 0.82 between rs112790520, a proxy for rs155327711, and rs9277341. The complete absence of HLA-DPA1 gene expression in cortex from fetal cortical samples, and near absence in infant cortex by RNA-Seq [34], is contrasted to later developmental epochs (child, teen, adult, and elderly) that show high levels of HLA-DPA1 (at FWER < 5%).

## 4. Discussion

This paper is the first report showing that alternative splicing processes HLA-DPA1 mRNA into multiple isoforms. The functional significance of these different isoforms of HLA-DPA1 in brain is not clear, but undoubtedly would alter the function of this gene since the IgG domain and transmembrane domain are both substantially altered, and both of these domains are highly conserved across vertebrates. Further work is required to functionally elaborate alterations of mRNA of 30–100 amino acids in the translated products.

Since HLA-DPA1 is part of a heteromeric complex, the alternatively spliced isoforms may act to inhibit complex formation and the presentation of antigens on the cell surface. Major histocompatibility complex class II molecules are expressed by immune antigen-presenting cells like B cells, dendritic cells, and monocytes/macrophages, and designed to stably bind and present fragments from exogenous proteins to the immune system. There are disorders involving MHC class II deficiency (MIM209920). Typically, patients with these types of disorders fail to fight off infections, have increased infections following viral, bacterial, and fungal exposures, and lack expression of different MHC Class II genes. Variations in the density of MHC class II molecules on antigen-presenting cells influence the intensity and the nature of T-cell response. Taken together, a lack of MHC Class II response results in less T-cell activation.

Patients failing to produce proper MHC II–peptide complexes do not mount efficient antibody responses to infection, and this is probably the underlying factor in differential hepatitis B viral resistance shown in association studies of HLA-DPA1 [23]. It was also shown that the same SNP (rs3077) was a strong eQTL for HLA-DPA1 expression in human liver samples (*p* = 10^−48^) [35], and LCLs [36]. This finding suggests that the expression of HLA-DPA1 is related to resistance to hepatitis B virus (HBV). However, we did not test association of HBV in our brain or blood samples in this experiment, but are suggesting that the rs3077 cis eQTL allele that increased expression of HLA-DPA1 was also associated with decreased HBV infection rates, thus indicating that decreased expression of the HLA-DPA1 gene could result in increased infection rates. The SNP used in our study (rs9277341) is in the same LD block (r2 > 0.8) as rs3077, and rs9277341 SNP is an eQTL across multiple brain regions for HLA-DPA1 expression as well shown in the present results. Further, shown in Figure 10 are the HLA-DPA1 SNP values for association with schizophrenia, demonstrating an individual SNP in this region with highly significant association (association *p* = 2.46 × 10^−10^). OR = 1.1956). Taken together, these genetic results and a strong eQTL in brain support a general susceptibility for SZ association with decreased HLA-DPA1 expression in multiple brain regions. In a survey of developmental cortical trajectory of gene expression, during fetal and infant brain development there is an absence of HLA-DPA1 expression in fetal brain, and very low levels in infant brain, contrasted to child, teen, adult, and elderly brains [34].

Besides this very strong eQTL at rs9277341 that we confirmed in brain and LCLs, there is an environmental factor that could perhaps also account for the findings of decreased expression, at least in LCLs of the MHC Class II molecules. This possibility is shown in the intriguing report that morphine and opioid agonists can reduce MHC II levels in circulating B-cells by 88% [36]. The underlying mechanism is the immunosuppressive effect of high cortisol levels induced by morphine. Circulating levels of cortisol are abnormally high in neuropsychiatric disorders [37,38], and could possibly account for the decreased expression of MHC Class II in the present LCL study. Although the cell lines were passaged and presumably independent of cortisol stimulation in the present study, there might be epigenetic changes associated near the HLA-DPA1 locus or in transcription factors that enhance HLA-DPA1 expression. A next logical step would be to study the interaction of cortisol suppression of HLA-DPA1 molecules by genotype interaction with the eQTL SNPs, and to determine the epigenetic landscape involved in the *ci*s eQTL effects observed in this study. Finally, we would need to determine more precisely the brain localization of HLA-DPA1 protein in brain. Previously, MHC Class I genes were shown to be localized to neurons and are essential for development and plasticity of neurons [24,39,40,41]. Little is known about expression of MHC Class II genes in brain, although we show that it is much lower in brain compared with PBMCs, transformed lymphocytes (EBV), and whole blood levels (Figure 9 A,B,C). Although it is not clear presently if neurons produce MHC Class II mRNA in human brain, MHC Class I and II are found on differential subpopulations of activated microglia in brain [42]. Expression of MHC Class II molecules are found in microglia using a general OX-6 antibody. To date, there is no specific study by immunohistochemistry with a specific monoclonal HLA-DPA1 antibody that has been conducted.

Since expression of MHC Class II appears to be reduced in brain and periphery, this might suggest an increased infection rate in patients with neuropsychiatric disorders. Although a higher rate of HIV in schizophrenia is reported, it is thought that the use of drugs and duration of untreated illness contributes to this increase [43]. Since there are literally thousands of immune-related genes, a follow-up paper is being planned to expand these current findings into the broader immune landscape using an independent array expression platform and analysis of immune-relevant upstream and downstream genes, as well as additional subjects, which also supports the present results for MHC Class II molecules [44].

In this study, we chose to use microarrays over next-generation sequencing (NGS). Both microarray and NGS technologies have their advantages and disadvantages; the selection depends upon the experimental goal. NGS has a wide range of applications and is particularly useful to investigate *de novo* mutations, new splice variants and non-coding RNA. NGS offers high sensitivity, high accuracy, and a broad dynamic range with no cross-hybridization. However, there are some limitations to NGS that make the use of microarrays a good alternative strategy. NGS has a higher cost with short reads that do not always allow the study of large splicing variants, chromosomal rearrangements or gene fusions like tiling arrays [45]. The analysis pipelines required for the NGS can be quite expensive and usually requires dedicated bioinformatics support to develop meaningful results.

On the other hand, microarrays have been used and accepted for the past three decades and have established analysis tools to speed the normalization and interpretation. Microarrays are powerful for analysis of known alternative splicing events [46]. Microarray technology is fast and a fairly reliable tool for applications involving gene expression, genotyping, and detection of known splicing variants. It requires, however, prior knowledge of the genome to design reliable probes with no cross hybridization in order to identify differences in transcript levels. In addition to gene and exon level expression, microarrays can also be used to investigate coding SNPs [47], but the detection ranges are limited and they require exceptionally high-quality mRNA. In the context of our experiments, microarrays produced reliable and replicable results concerning gene expression levels and led to the discovery of alternative splicing events of HLA-DPA1 transcripts.

## 5. Conclusions

Two genes in the MHC II complex were decreased in expression in brain and lymphoblast cell lines, most strongly in bipolar disorder and schizophrenia. This supports some shared immune alterations in brain for bipolar disorder and schizophrenia, and the observed strong genetic association of SZ to the MHC region [18] supports a role of HLA-DPA1 as a potential susceptibility gene. In MDD, there was decreased MHC II gene expression in brain, but not lymphoblast cell lines. In addition, a significant eQTL for HLA-DPA1 was validated in brain expression. The functions of the MHC Class II genes in the brain are unknown, and the role of these novel alternatively spliced HLA-DPA1 gene products found in the present study require further investigation. It has been shown that decreased HLA-DPA1 expression could be peripherally associated with HBV viral susceptibility. MHC Class II antigen presentation could act in brain to mark cells for immune attack by cells such as natural killer cells or macrophages that invade the blood−brain barrier. It has also been suggested that MHC and complement genes could alter synaptic plasticity such as pruning in brain circuits [24,39,40]; these findings may apply to our finding of a general reduction of MHC Class II expression in brain samples from patients with neuropsychiatric disorders. Our findings also support the long-held hypothesis that alterations in immune function are associated with the pathophysiology of psychiatric disorders.

Besides a purely genetic mechanism that might decrease MHC Class II expression, an increased cortisol level which has been previously shown to be elevated in psychiatric disorders could lower MHC Class II expression. This first report on MHC Class II genes across multiple brain regions opens the door for study of epigenetic and environmental regulatory effects. The strong eQTL in brain is in linkage disequilibrium with an irrefutable association with schizophrenia, demonstrating that alterations in immune expression in brain circuits during development may play a role in the pathophysiology of schizophrenia.

## Figures and Tables

**Table 1:** Experiment 1 anterior cingulate cortex samples were used in an exon array (Affymetrix HuEx 1.0 ST) from 9 bipolar disorder patients and 11 healthy controls for detection of alternative splicing.

Diagnosis	Sex	Age	pH	AFS	PMI	RIN-Anterior Cingulate
Bipolar Disorder	6 M; 3 F	46.22/17.3	6.89/0.2	0.00/0.0	24.17/8.7	7.97/1.0
Controls	11 M; 0 F	48.45/16.9	7.01/0.1	0.00/0.0	20.70/5.8	8.07/0.7
*p*-value for difference	0.074	0.77	0.09	‒	0.30	0.79

AFS (agonal factor score); PMI (postmortem interval); RIN (RNA integrity number).

**Table 2:** Five brain regions across three disorders (schizophrenia, bipolar disorder, major depressive disorder) and matched control subjects were analyzed by qPCR. The RNA integrity numbers (RINs) were available only for anterior cingulate (ACC) and dorsolateral prefrontal cortex (DLPFC). The same core group of subjects was used for analysis of five brain regions. Though there were some missing subjects in each brain region, the overlap with Experiment 1 subjects is shown (Supplementary Table 1).

Diagnosis	F (*N*)	M (*N*)	Total (*N*)	Mean and Std	Age	PMI	RIN-DLPFC	pH	RIN-Anterior Cingulate	AFS
BD	6	11	17	Mean (BD)	50.41	23.49	7.96	6.87	7.83	0.12
‒	‒	‒	‒	Std (BD)	16.39	8.56	0.49	0.15	0.95	0.33
C	9	40	49	Mean (C)	54.18	20.51	7.90	6.92	8.07	0.10
‒	‒	‒	‒	Std (C)	14.80	6.68	0.54	0.22	0.91	0.31
MDD	8	27	35	Mean (MDD)	45.69	24.89	7.93	6.92	8.16	0.06
‒	‒	‒	‒	Std (MDD)	14.13	6.42	0.58	0.26	0.81	0.24
SZ	4	11	15	Mean (SZ)	45.40	22.77	7.85	6.87	8.27	0.07
‒	Std (SZ)	8.72	10.66	1.66	0.21	0.51	0.26	Std (SZ)	8.72	10.66
Total	27	89	116	‒	‒	‒	‒	‒	‒	‒

F (female), M (male); AFS (agonal factor score); RIN (RNA integrity number); Std (standard deviation); PMI (postmortem interval); BD (bipolar disorder), C (control), MDD (major depressive disorder), SZ (schizophrenia).

**Table 3:** Lymphoblastic cell lines from 87 subjects were analyzed by qPCR for MHC Class II transcripts. The diagnosis and sex of those subjects are shown.

Diagnosis	Female	Male	Total
Bipolar Disorder	11	12	23
Control	10	12	22
Major Depressive Disorder	14	6	20
Schizophrenia	10	12	22
Total	45	42	87

**Table 4:** Individual HLA-DPA1 probesets from the Affymetrix human exon 1.0 ST array showing the *p*-value by *t*-test, and the fold change for bipolar disorder compared with controls in the anterior cingulate. The fold change shows that for differentially expressed probesets, the bipolar disorder samples were decreased compared to controls. The interaction effect between diagnosis and probesets was highly significant (*p* = 1.6 × 10^−6^).

Probeset Exon Array Number	Probeset Expression Difference (BD *vs* C) *p*-value	Fold Change (BD *vs* C)
2950330	0.429	−14%
2950331	0.004	−66%
2950332	0.003	−67%
2950333	0.011	−61%
2950334	0.114	−28%
2950335	0.965	−1%
2950336	0.009	−53%
2950338	0.069	−49%
2950340	0.066	−35%
2950341	0.021	−60%
2950342	0.045	−38%
2950343	0.002	−68%
2950345	0.080	−49%
2950346	0.012	−61%
2950347	0.174	−18%
2950348	0.007	−49%
2950349	0.353	−12%
2950351	0.422	−21%
2950352	0.353	−17%

**Table 5:** Additional genes assayed by qPCR to validate exon array results using cDNA from subjects shown in Table 2. The significant differences were shown in bold.

Gene Symbol	Exon Array		qPCR					
	*p*-value	Fold Change	*p* value	*p* value	*p* value	Fold Change	Fold Change	Fold Change
	BD *vs.* Control	BD *vs.* Control	BD *vs.* Control	MDD *vs.* Control	SZ *vs.* Control	BD *vs.* Control	MDD *vs.* Control	SZ *vs.* Control
*ZFP36*	**1.04 × 10^−5^**	0.54	**5.00 × 10^−3^**	0.907	**3.00 × 10^−2^**	0.40	1.04	0.57
*HLA-DRA1*	**4.42 × 10^−2^**	0.52	**8.00 × 10^−3^**	0.896	**8.00 × 10^−3^**	0.42	0.97	0.44
*ADAMTS1*	**2.30 × 10^−6^**	0.49	**1.00 × 10^−2^**	0.752	0.168	0.66	1.07	0.79
*RGS1*	**1.77 × 10^−4^**	0.47	**2.80 × 10^−2^**	0.274	**0.029**	0.26	0.64	0.25
*NR4A1*	**2.50 × 10^−9^**	0.45	**4.30 × 10^−2^**	0.667	0.249	0.33	1.16	0.62
*GSTM1*	**1.14 × 10^−2^**	0.49	**4.90 × 10^−2^**	0.089	0.136	0.25	0.35	0.41
*C10ORF4*	**1.24 × 10^−12^**	0.58	0.562	0.744	**3.10 × 10^−2^**	0.90	1.07	0.62
*TCF7L1*	**4.21 × 10^−5^**	0.76	0.187	0.122	**4.50×10^−2^**	0.81	0.78	0.68
*ESAM*	**6.81 × 10^−3^**	0.73	0.602	**0.038**	0.397	0.87	1.46	0.85
*FGFR3*	5.52 × 10^−1^	0.68	0.912	0.137	0.746	0.96	1.43	1.07

**Table 6:** Results of gene expression validation by qPCR in five brain regions using an ANCOVA model with region and diagnosis as main effects, and pH, sex, age, as covariates. Red *p*-values are significant.

Gene Symbol	Region	pH	Sex	Age	Diagnosis	Region by Diagnosis
*CD74*	2.71 × 10^−22^	8.20 × 10^−1^	6.71 × 10^−1^	5.01 × 10^−2^	3.61 × 10^−5^	5.84 × 10^−3^
*HLA-DRB1*	3.48 × 10^−1^	5.55 × 10^−2^	1.08 × 10^−3^	4.42 × 10^−5^	7.80 × 10^−2^	6.87 × 10^−1^
*HLA-DPA1*	7.93 × 10^−20^	3.80 × 10^−1^	2.77 × 10^−1^	1.79 × 10^−4^	1.73 × 10^−7^	2.55 × 10^−1^

**Table 7:** Five brain regions were analyzed by qPCR for schizophrenia, bipolar disorder, major depressive disorder and compared to matched control subjects. The ANCOVA results for each MHC II gene are shown for *p*-value and fold change comparisons for each neuropsychiatric group compared to controls. The significant differences were shown in bold.

Gene	Brain Region	*p*-value (Diagnosis)	*p*-value (BD *vs.* Control)	Fold Change (BD *vs.* Control)	*p*-value (MDD *vs.* Control)	Fold Change (MDD *vs.* Control)	*p*-value (SZ *vs.* Control)	Fold Change (SZ *vs.* Control)
CD74 qPCR	HC	**0.002**	**0.012**	−1.57	**0.001**	−1.70	**0.018**	−1.58
	Amy	**0.029**	0.078	−1.36	0.064	−1.27	**0.008**	−1.83
	ACC	**0.050**	**0.033**	−1.82	0.139	−1.30	0.471	1.14
	DLPFC	0.094	0.085	−1.45	0.177	−1.29	0.398	1.18
	NAcc	0.268	0.110	−1.33	0.278	−1.16	0.134	−1.32
HLA-DRB1 qPCR	Amy	0.181	0.907	−1.05	0.310	−1.40	0.036	−10.86
	HC	0.192	0.724	1.15	0.692	−1.16	0.047	−27.91
	DLPFC	0.259	0.445	1.46	0.422	1.38	0.168	−157.77
	ACC	0.314	0.654	1.21	0.767	−1.11	0.094	−4.57
	NAcc	0.326	0.632	−1.26	0.869	1.06	0.091	−4.61
HLA-DPA1 qPCR	HC	**0.002**	**0.024**	−1.54	**0.0004**	−1.81	**0.031**	−1.56
	DLPFC	**0.002**	**0.0004**	−2.69	**0.013**	−1.53	0.176	−1.33
	ACC	0.119	**0.036**	−1.65	0.068	−1.34	0.405	−1.18
	Amy	0.239	0.269	−1.34	0.325	−1.21	0.057	−2.01
	NAcc	0.245	0.190	−1.29	0.150	−1.25	0.096	−1.43

**Table 8:** HLA-DPA1 expression evaluated with main effects of genotype (rs9277341), brain region, and diagnosis by ANCOVA. The effect of diagnosis was significant as shown.

Group	Mean	LSMean	StdErr	*p*-value (*vs*. Control)	Fold Change (*vs*. Control)
Control	0.430	0.394	0.021		
MDD	0.292	0.284	0.015	6.72 × 10^−6^	−1.39
Schizophrenia	0.268	0.278	0.026	4.32 × 10^−4^	−1.42
Bipolar Disorder	0.290	0.270	0.030	4.57 × 10^−5^	−1.46

**Table 9:** HLA-DPA1 expression evaluated with main effects of genotype, region, and diagnosis by ANCOVA. The effect of diagnosis was significant as shown.

(Region)	(Diagnosis)	(Agonal_factor)	(pH)	(Age)	(rs9277341)	(Region By rs9277341)
3.15 × 10^−17^	1.68 × 10^−6^	0.0008	0.21	6.56 × 10^−5^	0.0002	0.89

**Table 10:** The sequence of four subjects shows full-length amplicons of exons 2, 3, and 4 (512–515 bp), and truncated versions of exons 2, 3, 4 (215–400 bp). The dominant form of HLA-DPA1 was a full-length product, but several alternatively spliced isoforms were found (Figure 4 A-H). For example, sequences shown in UCSC Tracks (Figure 4 A–H), show truncation of exons 2, 3, and 4, thus suggesting different cryptic splice sites that occur and subsequently produce variable missing sequences ranging from 104–242 bp.

Subject (Diagnosis)	Sequenced Band (Same Numbers as UCSC Track)	Missing Position in NM_033554.3	Missing Sequences	Missing In
11 (Bipolar)	HLA-DPA1_4-2_1	479–641	163	exon 3
5 (Control)	HLA-DPA1_4-2_3	364–654	291	exon 2, 3
5 (Control)	HLA-DPA1_4-2_4	615–759	145	exon 3, 4
5 (Control)	HLA-DPA1_4-2_5	655–758	104	exon 3, 4
3 (Control)	HLA-DPA1_4-2_7	433–640	208	exon 2, 3
3 (Control)	HLA-DPA1_4-2_8	433–640	208	exon 2, 3
6 (Control)	HLA-DPA1_4-2_10	361–602	242	exon 2, 3
6 (Control)	HLA-DPA1_4-2_11	667–782	116	exon 3, 4

**Table 11:** The LCLs (*N* = 87, Table 3) were regrown and mRNA for each transcript was measured with qPCR assays. The numbers in bold show the significant fold-change and *p*-values for each of the comparisons to the control group.

Class II mRNA	Fold Change			*p*-value		
	BD	MDD	SZ	BD	MDD	SZ
CD74	**0.54**	1.25	0.62	**0.036**	0.583	0.156
HLA-DPA1	**0.53**	1.11	**0.50**	**0.037**	0.812	**0.057**
HLA-DRB1	0.92	5.43	1.26	0.956	0.208	0.870

**Table 12:** Summary of blood−brain comparisons by exon array for evaluation of differential expression between different tissues for MHC Class II mRNAs.

Tissue (Diagnosis)	Count of Tissue
Anterior Cingulate Cortex	20
BD	9
C	11
Cerebellum	5
BD	1
C	2
MDD	2
Occipital	4
C	4
P0 Pre-transformed PBMC	8
C	8
P1 Passage PBMC EBV Transformed	7
C	7
P2 PassagePBMC EBV Transformed	7
C	7
PBMC-Trizol-Fresh	71
C	71
PBMC-Trizol-Postmortem	5
BD	1
C	3
MDD	1
Transformed, Multiple Passages	32
Sz	32
Transformed, Multiple Passages—low glucose	10
C	5
Sz	5
Transformed, Multiple Passages—normal glucose	10
C	5
Sz	5
Whole blood_Tempus	8
C	8
Grand Total	187

## Supplementary Material

The following are available online at www.mdpi.com/2076-3905/5/1/6/s1.
