# Supplementary material for: Quantitative Trait Locus and Brain Expression of HLA-DPA1 Offers Evidence of Shared Immune Alterations in Psychiatric Disorders
Source: Microarrays (Basel). 2016 Mar 7;5(1):6. doi: 10.3390/microarrays5010006 (PMC4795482; doi:10.3390/microarrays5010006)
Supplement: Supplementary file 1 [file microarrays-05-00006-s001.zip › 19. Supplementary Material/Supplementary Methods.pdf]

Supplementary Methods: Quantitative Trait Locus and Brain Expression of HLA-DPA1 Offers Evidence of Shared Immune Alterations in Psychiatric Disorders

Ling Z. Morgan, Brandi Rollins, Adolfo Sequeira, William Byerley, Lynn E. DeLisi, Alan F. Schatzberg, Jack D. Barchas, Richard M. Myers, Stanley J. Watson, Huda Akil, William E. Bunney Jr and Marquis P. Vawter

1. Equation implemented in Partek Genomics for calculation of the interaction between probeset and diagnosis:

$$y = \mu + D + S + E + (D * E) + \sum \varepsilon$$

y: expression of a gene; μ: average expression of the gene; D: diagnosis effect; S: subject to subject effect (this is a random effect); E: exon-to-exon effect (probeset differences independent to diagnosis); D * E: an exon (probesets) expresses differently in different diagnosis (alt-splicing dependent on diagnosis type); ε: is the error term

2. The design of the Affymetrix exon array 1.0 ST is from the manufacturer's website.

Design Statistics Summary	
Probe Sets	1.4 million #
Exon Clusters	> 1 million
Supported by Putative Full-Length mRNA	289,961 probe sets
Supported by Ensembl Transcripts	306,583 probe sets
Supported by EST	665,175 probe sets
Supported by Mouse or Rat mRNA	220,262 probe sets
Supported by Gene Prediction	883,105 probe sets
Probe Selection Region	Along the entire length of the transcripts
Probes/Probe Selection Region	4 [1]
Background Subtraction Strategy	Median intensity of up to 1,000 background probes with the same GC content
Total Features Per Array	>5,500,000
Interrogated Strand	Sense [2]

[1] About 10 percent of the exon probe sets have fewer than four probes due to the probe selection region length and sequence constraints; [2] The probes tiled on the array are designed in the anti-sense orientation, requiring sense-strand labeled targets to be hybridized to the array. By convention, the array is called ST array representing the necessity of using sense targets (the labeled samples to be hybridized to the array). # We eliminated probes with common SNPs following method of Gamazon *et al.*, 2010 [30], that reduced the probeset count by ~350,000 probesets. Further we report only findings that are associated with full-length mRNA and have coverage by at least 2 probesets. This reduced the total probesets analysis to 11, 807genes represented with 230,659 probesets.

3. Primer Table.

Gene	Primers			
	**Forward**	**Reverse**	**Exon array Probeset**	**Exon**
HLA-DPA1				
qPCR	CCCTGAGGTG ACCGTGTTTC	GTCAATGTGGC AGATGAGGGT	RP inside of probeset 2950343	FP Exon3, RP Exon3
qPCR – No SNP	ATCCAGCGT TCCAACCAC	AATGTGGCAGA TGAGGGTGT	RP overlap of probeset 2950343	FP Exon2, RP Exon3
Sequencing	TGGACAAGAA GGAGACCGTCT	TTTATGATGA GGACGGTGCC		FP Exon2, RP Exon4
96 CR transformed LCLs	Same as qPCR-No SNP	Same as qPCR-No SNP	Same as qPCR-No SNP	Same as qPCR-No SNP
rs9469341 QPCR &sequenceing	ATGTGGTCAT CTGCATGGAA	AGTGTGGTGG GTGCCTGTA		Intron4, Intron 4
rs9277341 QPCR &sequenceing	TCAGGGAAG ACAGCCTGACCGG	CCTGCACCTGCC ACTAATGACTCT		Intron1, Intron 1
HLA-DRB1				
qPCR	TTTGCCTAAA CCGTATGGCCT	TGTGTTTGTCATAC AGGGTGAATACA	RP overlap of probeset 4048279	Both Exon6
qPCR – No SNP				
Sequencing				
96 CR transformed LCLs	Same as qPCR	Same as qPCR	Same as qPCR	Same as qPCR
CD74				
qPCR	GCTCTTTCGT CACCCTTGGAC	CCTTGCTGCAT TGTTATCTGCT	FP and RP both inside of probeset 2881380	Both Exon5
qPCR – No SNP				
Sequencing				
96 CR transformed LCLs	Same as qPCR	Same as qPCR	Same as qPCR	Same as qPCR

4. Comparison of Exon microarray and qPCR genes from anterior cingulate.

Gene symbols	ExonArray		qPCR					
	p-value	Fold Change	*p* value			Fold Change		
	**BD *vs* Control**	**BD *vs* Control**	**BD *vs* Control**	**MD *vs.* Control**	**Schiz *vs.* Control**	**BD *vs* Control**	**MD *vs* Control**	**Schiz *vs* Control**
ZFP36	**1.04×10^{-5}**	0.54	**0.005**	0.907	**0.030**	0.40	1.04	0.57
HLA-DRA1	**4.42×10^{-2}**	0.52	**0.008**	0.896	**0.008**	0.42	0.97	0.44
ADAMTS1	**2.30×10^{-6}**	0.49	**0.010**	0.752	0.168	0.66	1.07	0.79
RGS1	**1.77×10^{-4}**	0.47	**0.028**	0.274	**0.029**	0.26	0.64	0.25
NR4A1	**2.50×10^{-9}**	0.45	**0.043**	0.667	0.249	0.33	1.16	0.62
GSTM1	**1.14×10^{-2}**	0.49	**0.049**	0.089	0.136	0.25	0.35	0.41
C10ORF4	**1.24×10^{-12}**	0.58	0.562	0.744	**0.031**	0.90	1.07	0.62
TCF7L1	**4.21×10^{-5}**	0.76	0.187	0.122	**0.045**	0.81	0.78	0.68
ESAM	**6.81×10^{-3}**	0.73	0.602	**0.038**	0.397	0.87	1.46	0.85
FGFR3	5.52×10^{-1}	0.68	0.912	0.137	0.746	0.96	1.43	1.07

The *p*-value numbers in bold show the significant differences for each comparison.
